# Impact of human papillomavirus on head and neck squamous cell cancers in Gabon

**DOI:** 10.1186/s13027-015-0036-7

**Published:** 2015-11-09

**Authors:** Labouba Ingrid, Bertolus Chloé, Koumakpayi Ismail Hervé, Belembaogo Ernest, Miloundja Jérôme, Berthet Nicolas

**Affiliations:** Department of Zoonosis and Emerging Diseases, Centre International de Recherches Médicales de Franceville – Gabon (CIRMF – GABON), B.P. 769 Franceville, Gabon; Department of Maxillofacial surgery, AP-HP, Hôpital Pitié-Salpêtrière, Paris, F-75013 France; UPMC, Université Paris 06, F-75005 Paris, France; Cancer Institute of Libreville, Laboratory of Tumor Biology, Libreville, Gabon; Department of ENT, Omar Bongo Ondimba Army Instruction Hospital, Libreville, Gabon; Centre National de la Recherche Scientifique, UMR3569, 25 rue du docteurRoux, 75724 Paris, France

**Keywords:** HPV, HNSCC, Oropharynx, Oral cavity, Tobacco, Alcohol, Middle Africa, Biomarker, Vaccination

## Abstract

Head and neck squamous cell cancers are among the most aggressive. Their incidence and mortality rates are relatively lower in Middle Africa than worldwide, but in Gabon, these rates tend to be 2–3 fold higher than in neighboring countries. The main risk factors are alcohol and tobacco consumption. However, in the last decades, there was cumulated evidence that human papillomaviruses were a significant risk factor, particularly for oropharyngeal squamous cell cancer. In Gabon, as elsewhere in Africa, assessment of these 3 risk factors need to be improved to determine their respective role in the development of head and neck squamous cell cancers. The potential differences in alcohol/tobacco consumption habits as well as in infectious ecology between developing and developed countries can make it difficult to transpose current data on this issue. Determining the respective role of alcohol/tobacco consumption and human papillomaviruses in the development of head and neck squamous cell cancers is crucial for the management of these cancers that could become a serious public health issue in Gabon. Human papillomaviruses are not only a risk factor but also a biomarker with promising clinical potential for the follow-up of head and neck squamous cell cancers potentially able to select an adequate treatment. Then, assessing the epidemiological impact of human papillomaviruses in Gabon and in all of Africa would prove useful for the clinical follow-up of head and neck squamous cell cancers, and would also provide essential data to plan a global prevention strategy against head and neck squamous cell cancers due to human papillomaviruses.

## Background

Head and neck (excluding nasopharyngeal) cancers, most frequently squamous cell carcinoma, are among the most aggressive worldwide with 599,637 new cases diagnosed and 324,834 associated-deaths in 2012 [1, 2]. We study more specifically oral cavity (OCSCC) and oropharynx (OPSCC) squamous cell carcinomas, still grouped despite the important anatomic and histological distinctions [3]. Their main risk factors, involved in 75–80 % of cases, are tobacco and alcohol [4, 5]. Human papillomaviruses (HPV) are also a relevant risk factor for head and neck squamous cell carcinomas (HNSCC), mainly OPSCC [6, 7]. The relationship between HNSCC and HPV has been well demonstrated in the United States of America (U.S.A.) and in Europe where up to 60 % of OPSCC could be attributed to HPV [8–10]. This raises the issue of HPV vaccination to prevent HNSCC as well as HPV-induced cervical cancers.

## HNSCC risk factors in Middle Africa

Alcohol and tobacco consumption habits vary according to regions (Table 1) and make it difficult to extrapolate their impact on HNSCC from developed to developing countries. Similarly, differences in the infectious ecology between developed and developing countries complicate transposing the documented involvement of HPV in OPSCC to Africa where the profile of HPV infection is yet to be determined. Furthermore, the impact of HPV, as well as of alcohol and tobacco on HNSCC in Africa, needs to be more extensively studied before defining the best prevention strategy for HNSCC.

**Table 1:** Alcohol and tobacco consumption in the U.S.A, France, and Gabon [13, 14]

	Average alcohol consumption^a^			2010 Alcohol consumption (drinkers only)^a^	2010 prevalence of binge drinking^b^ (%)		Estimated prevalence of cigarette smokers (%)^c^	
	2003 – 2005	2008 – 2010	Change		Population	Drinkers only	Current	Daily
United States of America	9.5	9.2	→	13.3	16.9	24.5	18.0	14.3
*WHO region of Americas*	*9.2*	*8.4*	_					
France	13.4	12.2	→	12.9	29.4	31	22.8	20.1
*WHO European region*	*11.9*	*10.9*	*_*					
Gabon	8.8	10.9	↗	26.5	5.3	12.9	9.0	7.0
*WHO African region*	*6.2*	*6* *.0*	*_*					

^a^Alcohol per capita consumption (in liter per capita of pure alcohol) among 15+ population

^b^Consumed at least 60 g or more of pure alcohol on at least one occasion in the past

^c^2013 data for U.S.A and France except for Gabon (last update in 2011)

**Fig. 1:**
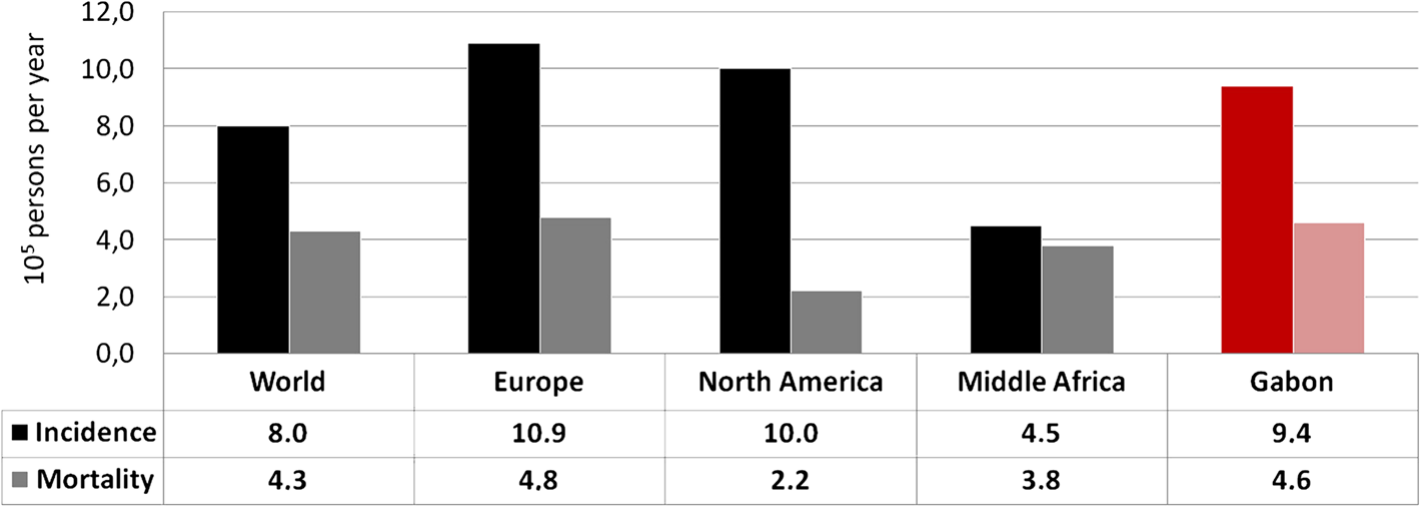
Estimated age-standardized Incidence (ASIR) and Mortality (ASMR) rates of HNSCC in 2012. Graphic representations and associated numbers (10^5^ persons per year) of incidence and mortality rates of all HNSCC worldwide and in various regions. Bars for Gabon, country of interest here, are in red/pink whereas they are black/grey for Europe, Northern America, Middle Africa and worldwide (as World). Dark bars (red for Gabon and black for other regions) represent the incidence rates and the light ones (pink for Gabon and grey for other regions) represent the mortality rates. (Source: GLOBOCAN 2012) [1, 2]

In Middle Africa, the incidence of HNSCC (100,000 new cases/year) is lower than the worldwide rate (respectively 4.5 versus 8.0), except in Gabon where reported rates are 2–3 fold higher (9.4) (Fig. 1) [1, 11, 12]. However, it is doubtful that these numbers perfectly reflect the true HNSCC rates in Middle Africa and in Gabon. As elsewhere in Africa, cancer registration is not routinely implemented yet in the healthcare institutions managing cancers. Attributing the differences between Gabon and the rest of Middle Africa to a true susceptibility of the Gabonese population or simply to a better screening/registering/follow-up of HNSCC is a real challenge. In any case, the main issue remains: *how to manage HNSCC, which could become a real public health problem, in Gabon?*

## Tobacco and alcohol consumption in Gabon

The epidemiology of tobacco and alcohol consumption in Gabon is still poorly documented and assessing their impact represents is a real challenge in developing countries. Tobacco and alcohol prevention programs have induced a significant decrease of HNSCC incidence in developed countries [10]. Similar programs have not been implemented yet in Africa where tobacco and alcohol abuse are increasing [13, 14].

The total alcohol consumption in the Gabonese adult population (15+) was 26.50 l per capita of pure alcohol in 2010 (Table 1) compared to 7.90 l in 2005 [15]. The trend in Gabon was clearly increasing from 2003 to 2010 unlike in the U.S.A and in France where it remained stable during the same period (Table 1) [14, 15]. This data, mainly based on import/export economic values, does not take into account indigenous alcoholic products commonly used in local habits. The alcohol consumption in Gabon as well as its true impact on HNSCC could thus be underestimated.

The prevalence of current smokers in Gabon in 2011 was estimated at 15.0 % of men and 2.0 % of women in a population 15 years of age or more (15+) for a global rate of 9.0 % in this population. These rates are lower for daily smokers: global rate of 7.0 % with 12.0 % of men and 1.0 % of women (Table 1). The estimated prevalence of current smokers was respectively 18.0 and 22.8 % in developed countries such as the U.S.A and France in 2013. The rate of smokers was much lower in Gabon (9.0 %) even though increasing (Table 1) as illustrated by 2 studies on Gabonese teenagers [13]. 10.9 % of the population 14–22 years of age smoked in 2007 [16], compared to 21.5 % in the population 10 – 19 years of age in 2011 [17].

The increasing consumption of tobacco and alcohol has become a matter of public health concern in Gabon and requires to be assessed accurately to better understand its impact on HNSCC in the Gabonese population. The example of betel in India and HNSCC perfectly illustrates the importance of local habits. The consumption of betel was documented as a major risk factor for OCSCC in India where tobacco and alcohol consumption is moderate [18].

## HPV and cancers in Gabon: screening and vaccination?

In Africa, most efforts in the prevention of HPV related cancer are focused on CC, associated with HPV infection in 80 % of cases. Cervical cancer (CC) is the most frequently diagnosed and the main cancer-related cause of death in the female population in Africa as well as in Gabon, [4, 11, 12]. Nevertheless, in Gabon, systematic HPV screening has not been integrated yet in the cervical neoplasia detection process. HPV DNA screening relies essentially on PCR with specific/degenerated primer systems or on hybrid capture systems largely used in developed countries. These systems are still too expensive to implement systematic HPV DNA screening in Africa, [19]. However, an immunohistochemical approach could be an affordable alternative with p16 staining as marker of active HPV infection. The public health in Africa and Gabon is currently working on the implementation of efficient programs of early CC screening that would group cervix cytology and HPV detection. Finally, this also should allow for developing prevention strategies by integrating systematic anti-HPV vaccination in the young female population. These efforts to prevent CC raise the issue of other HPV-attributable cancers such as HNSCC.

OPSCC are the most frequent HPV-attributable HNSCC [20, 21]. The authors of a recent study on a West African cohort reported that, in Senegal, the low prevalence (3.4 %) of HPV in HNSCC lesions in 117 patients suggested no significant association with HPV infection, independently of upper airways anatomic sites. However, it is important to underline the small number of OPSCC included (only 4/117 cases) which could lead to underestimate the true impact of HPV in HNSCC [22]. To our knowledge, no team has ever evaluated the cause/effect link between HPV and HNSCC yet, particularly for OCSCC and OPSCC, in Middle Africa including in Gabon. Furthermore, the prevalence of HPV in HNSCC as well as the main HPV types remain undocumented. Nevertheless, this point is essential to evaluate beforehand the potential impact of anti-HPV vaccination in preventing HNSCC. Bi- and tetravalent vaccines currently under clinical trials have been developed against the main genital HPV types. A new generation nonavalent vaccine could enlarge the anti-HPV spectrum, however, it is still necessary to document whether HPV in the upper airways could be targeted [23]. Furthermore, the HPV status would have an effective prognostic value on the treatment response and on the survival of HNSCC patients [24, 25]. Kreimer *et al.* reported that even the presence of specific HPV 16/18 antibodies could predict HNSCC development, stressing the potential clinical value of HPV status [26]. Thus, the HPV status appears as a promising tool: **i)** to plan an adapted prevention strategy, **ii)** as a biomarker for a better follow-up of HNSCC patients, particularly in non-smokers and non-alcohol consumers [21].

## Conclusion

HNSCC is becoming a matter of public health concern in Gabon. It is necessary to control the main risk factors for their prevention: tobacco, alcohol and HPV. First, it is imperative to determine accurately the rate of these risk factors in Gabon. Therefore, local smoking and drinking habits should be documented to implement adapted programs to raise the awareness of the Gabonese population. Moreover, extending HPV research to the whole Gabonese population is also essential. Numerous ongoing studies are focused on the female population to determine the HPV infection profile in a context of CC. Extending this research to HNSCC would be a valuable move to assess the HPV relative risk. This would ultimately allow for defining the global epidemiological impact of HPV to elaborate an effective vaccine anti-HPV strategy thus preventing the occurrence of HPV-attributable HSNCC.

## Abbreviations

15+: Fifteen years and more
HNSCC: Head and neck squamous cell carcinomas
HPV: Human papillomaviruses
OCSCC: Oral cavity squamous cell carcinomas
OPSCC: Oropharyngeal squamous cell carcinomas
U.S.A.: United States of America
WHO: World Health Organization
