# Erratum to: Impact of human papillomavirus on head and neck squamous cell cancers in Gabon

**DOI:** 10.1186/s13027-016-0054-0

**Published:** 2016-02-04

**Authors:** Ingrid Labouba, Chloé Bertolus, Hervé I. Koumakpayi, Ernest Belembaogo, Jérôme Miloundja, Nicolas Berthet

**Affiliations:** Department of Zoonosis and Emerging Diseases, Centre International de Recherches Médicales de Franceville – Gabon (CIRMF – GABON), Franceville, B.P. 769 Gabon; Department of Maxillofacial surgery, AP-HP, Hôpital Pitié-Salpêtrière, Paris, F-75013 France; UPMC, Université Paris 06, Paris, F-75005 France; Cancer Institute of Libreville, Laboratory of Tumor Biology, Libreville, Gabon; Department of ENT, Omar Bongo Ondimba Army Instruction Hospital, Libreville, Gabon; Centre National de la Recherche Scientifique, UMR3569, 25 rue du docteurRoux, Paris, 75724 France

After publication of this article [1], the authors noticed their names had been incorrectly reverted. The authors’ names were therefore incorrectly presented as: Labouba Ingrid, Bertolus Chloé, Koumakpayi Ismail Hervé, Belembaogo Ernest, Miloundja Jérôme and Berthet Nicolas.

The correct presentations of the authors’ names are included in the author list of this erratum and have also been updated in the original article [1].
